# Isolation of anti-extra-cellular vesicle single-domain antibodies by direct panning on vesicle-enriched fractions

**DOI:** 10.1186/s12934-017-0856-9

**Published:** 2018-01-13

**Authors:** Milica Popovic, Elisa Mazzega, Barbara Toffoletto, Ario de Marco

**Affiliations:** 1 Faculty of Chemistry, Department of Biochemistry, University of Belgrade, Studentski trg 12-16, 11000 Belgrade, Serbia; 2 Laboratory for Environmental and Life Sciences, University of Nova Gorica, Glavni Trg 8-SI-5271, Vipava, Slovenia; 3 Azienda Sanitaria Universitaria Integrata di Udine–Istituto di Anatomia Patologica, Udine, Italy

**Keywords:** Nanobodies, Extracellular vesicles, Panning strategy, Exosomes, Monolith chromatography

## Abstract

**Background:**

The thorough understanding of the physiological and pathological processes mediated by extracellular vesicles (EVs) is challenged by purification methods which are cumbersome, not reproducible, or insufficient to yield homogeneous material. Chromatography based on both ion-exchange and immune-capture can represent an effective method to improve EV purification and successive analysis.

**Methods:**

Cell culture supernatant was used as a model sample for assessing the capacity of anion-exchange chromatography to separate distinct EV fractions and to isolate nanobodies by direct panning on whole EVs to recover binders specific for the native conformation of EV-surface epitopes and suitable to develop EV immune-capture reagents.

**Results:**

Anion-exchange chromatography of cell culture supernatant separated distinct protein-containing fractions and all of them were positive for CD9, a biomarker associated to some EVs. This suggested the existence of several EV fractions but did not help in separating EVs from other contaminants. We further isolated several nanobodies instrumental for implementing immune-affinity protocols. These were able to immobilize EVs from both cell culture supernatant and biological samples, to be used in ELISA, flow-cytometry, and immune-purification.

**Conclusions:**

Here we report the first successful isolation of anti-EV nanobodies for the use in immunoaffinity-based EV capture by panning a phage library directly on partially purified EVs. This achievement paves the way for the application of direct EV panning for the discovery of novel antibody-vesicle surface biomarker pairs and represents the preliminary requirement for the development of selective immune-capture that, in combination with anion-exchange chromatography, can simplify the systematic stratification of EV sub-populations and their individual characterization.

**Electronic supplementary material:**

The online version of this article (10.1186/s12934-017-0856-9) contains supplementary material, which is available to authorized users.

## Background

Extracellular vesicles (EVs) have been identified in many biological fluids such as blood, urine, cerebrospinal fluid, milk, ascites. The present scientific interest for EVs stems from the discovery that they play a crucial role in paracrine and long-distance cell–cell communication in physiological and pathological conditions as different as coagulation, inflammation, regenerative and differentiation processes, immune system modulation, tumor growth and metastasis [1–7]. EVs specifically deliver their cargos thanks to surface displayed proteins that have affinity for target-cell receptors [8]. Their stable lipid bilayer forms a relatively large internal volume in which regulatory messengers (nucleic acids, lipids, proteins, and metabolites) are protected during transport and finally released by internalization or direct fusion with target cell membranes [9, 10]. Since EVs are easily accessible in biological fluids, they are evaluated as diagnostic and prognostic biomarkers in liquid biopsy assays [11, 12]. However, this perspective will be realized only after having established reliable and reproducible purification methods for eliminating contaminants and, possibly, discriminating among EV sub-classes carrying distinct molecular information [13]. Comparative surveys of EV purification methodologies indicate that EVs can be obtained highly pure by a combination of density gradient ultracentrifugation and size-exclusion chromatography or ultrafiltration, but fractionations of EV subclasses is mostly dependent on affinity techniques [14–20].

Although size exclusion chromatography is a standard step during EV purification, other chromatographic techniques have not been generally assessed. The major reason is the large EV diameter that could lead to rapid clogging of resin-based columns. Nevertheless, ion-exchange chromatography can be used to recover EVs, as recently demonstrated with different anion exchange setups [21–23]. Particularly interesting are monolith columns since their structural stability and the possibility to obtain pores of variable diameter make them compatible with EV immobilization and recovery [23]. By using such material, there is the chance to exploit the difference in EV charges and to elute separately distinct EV classes. Sub-population specificity could be improved by coupling orthogonal chromatographic separation methods such as ion-exchange and immunoaffinity. The limiting factor for the development of immune-based capture is the identification of reliable and possibly inexpensive binders specific for EV epitopes, optimally ones that are able to discriminate among vesicle sub-groups. Conventional antibodies—generally poly- and monoclonal antibodies of the IgG class—have been successfully used for immune-affinity purification of EV sub-groups [24]. Nevertheless, some of their characteristics are unwanted, such as the quality differences among lots, their long isolation procedure and elevated production costs, their genetic instability, and the low homogeneity after labelling/functionalization [25, 26]. Single-domain antibodies (VHHs, nanobodies) have reduced mass (14 kDa in comparison to the 150 kDa of an IgG), are structurally stable, simple to engineer and label at specific residues, inexpensive to produce in bacteria and their clonality remains constant over the time [27]. These characteristics make them appreciated and effective reagents in different applications such as oncology, infectious, inflammatory, and neurodegenerative diseases [28]. A further advantage is that large pre-immune libraries are available and can be directly panned against both soluble antigens and whole cells [29–33]. We speculated that also EVs could be used as material for direct panning and that this approach would represent an advantage because it is rapid and would enable the isolation of binders for the native conformation of the antigen exposed on the EV surface. This is critical because it has been reported that the antigen present on EVs can be slightly modified with respect to that expressed in the original cell [34]. In this paper we demonstrate: (i) the possibility to separate EV-containing fractions according to their retention in a monolith anion-exchange column; (ii) the feasibility of direct panning on EV-enriched fraction to isolate nanobodies that recognize membrane epitopes on such vesicles. Now that the technical conditions have been established, the method will enable to isolate further binders for different antigens and this antibody pool will allow for more detailed characterization of EV fractions. In combination with anionic-exchange chromatography, immune-capture will contribute to the stratification of EV populations.

## Results

### Anion-exchange chromatography for EV fractionation

**Fig. 1:**
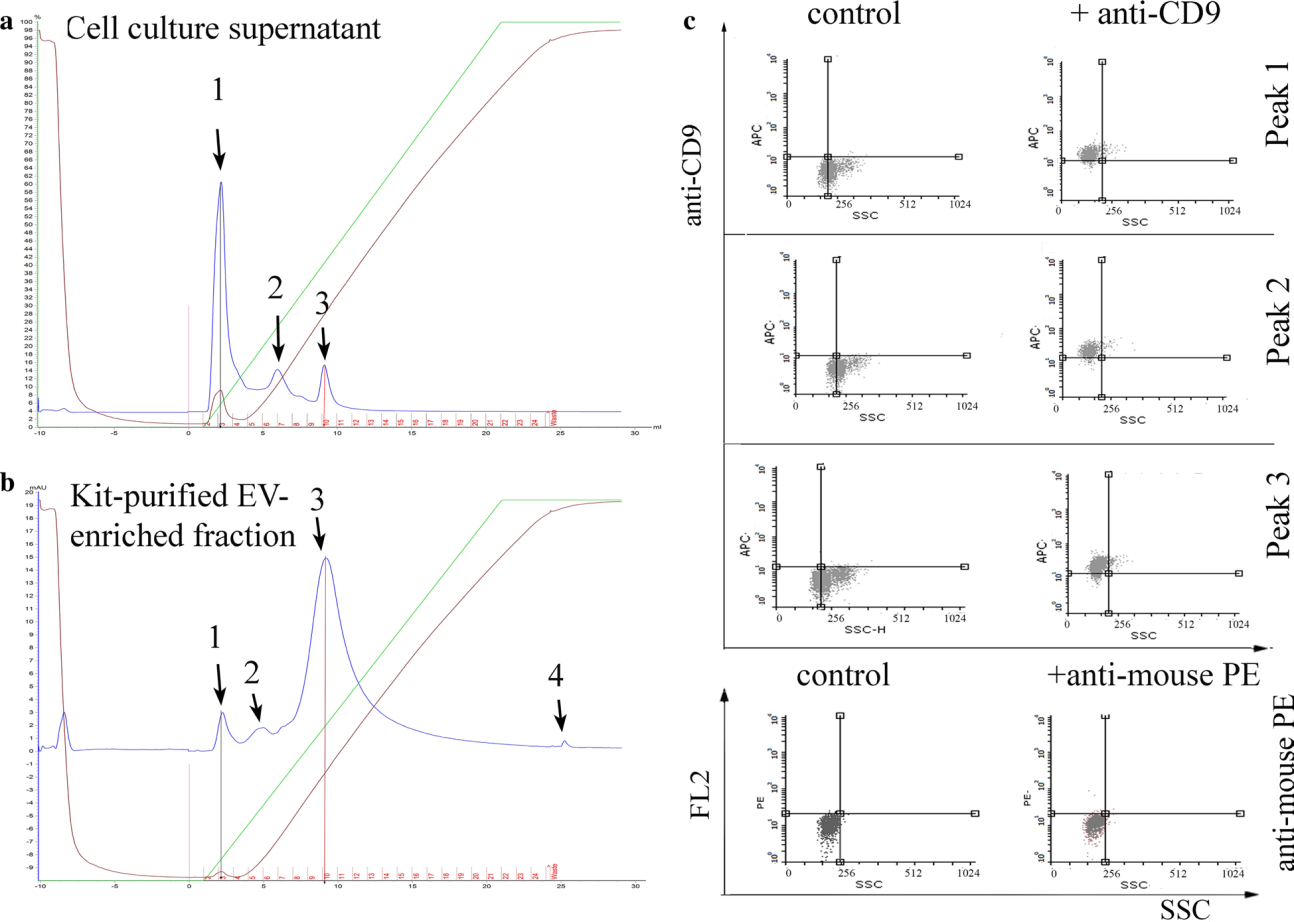
Chromatographic separation of EV-containing fractions present in cell culture media. Proteins present in SKBR3 cell culture supernatant (**a**) and in the kit-purified EV-enriched fraction from the same supernatant (**b**) were separated using a large-pore anion-exchange monolith column. Three and four independent elution fractions were detected, respectively, and fractions corresponding to peaks 1 and 3 (black and red bars) eluted at coincident salt concentrations in the two samples. All the three fractions separated from the cell-culture supernatant sample were analyzed by flow-cytometry and resulted positive for the EV marker CD9 (**c**). The irrelevant anti-mouse PE-labeled antibody was used to evaluate possible unspecific interactions between EV fractions and antibodies during flow-cytometry. Gates were set according to the values of autofluorescence of naked beads—not coated with EVs

Liquid chromatography performed using a monolith anion-exchange column and applied to medium from cancer cell culture supernatant resulted in the separation of three distinct elution peaks (Fig. 1a). Apparently, all the three fractions contained EVs because they were positive for CD9 when analyzed by flow cytometry (Fig. 1). An EV enrichment step obtained submitting the cell culture supernatant to ultrafiltration and successive precipitation by means of the Total Exosome Isolation kit [35] induced a significant modification of the chromatographic elution profile (Fig. 1b). Four peaks were detected, with peaks 1 and 3 sharing the same retention volume of the homologous peaks eluted from the untreated cell culture supernatant. Peaks 2 and 4 have no direct correspondence in the elution profile from the untreated cell culture supernatant (the retention time of peak 2 differed in the two samples, peak 4 was detected only in the second sample) but were also positive for CD9 (Additional file 1: Figure S1). Strikingly, the precipitation step significantly reduced the protein content of peak 1 (from 60 to 3 mAU), while the protein content of peak 3 remained constant (15 mAU). The thorough characterization of the molecular content of the different fractions will be the object of a further project, but these preliminary results already enabled to establish that EV surface charge is sufficient to separate distinct sub-classes. Their further characterization relies on the availability of another orthogonal purification method, such as immunopurification exploiting antibodies for specific surface antigens.

### Panning of nanobodies on EV-enriched fractions

**Fig. 2:**
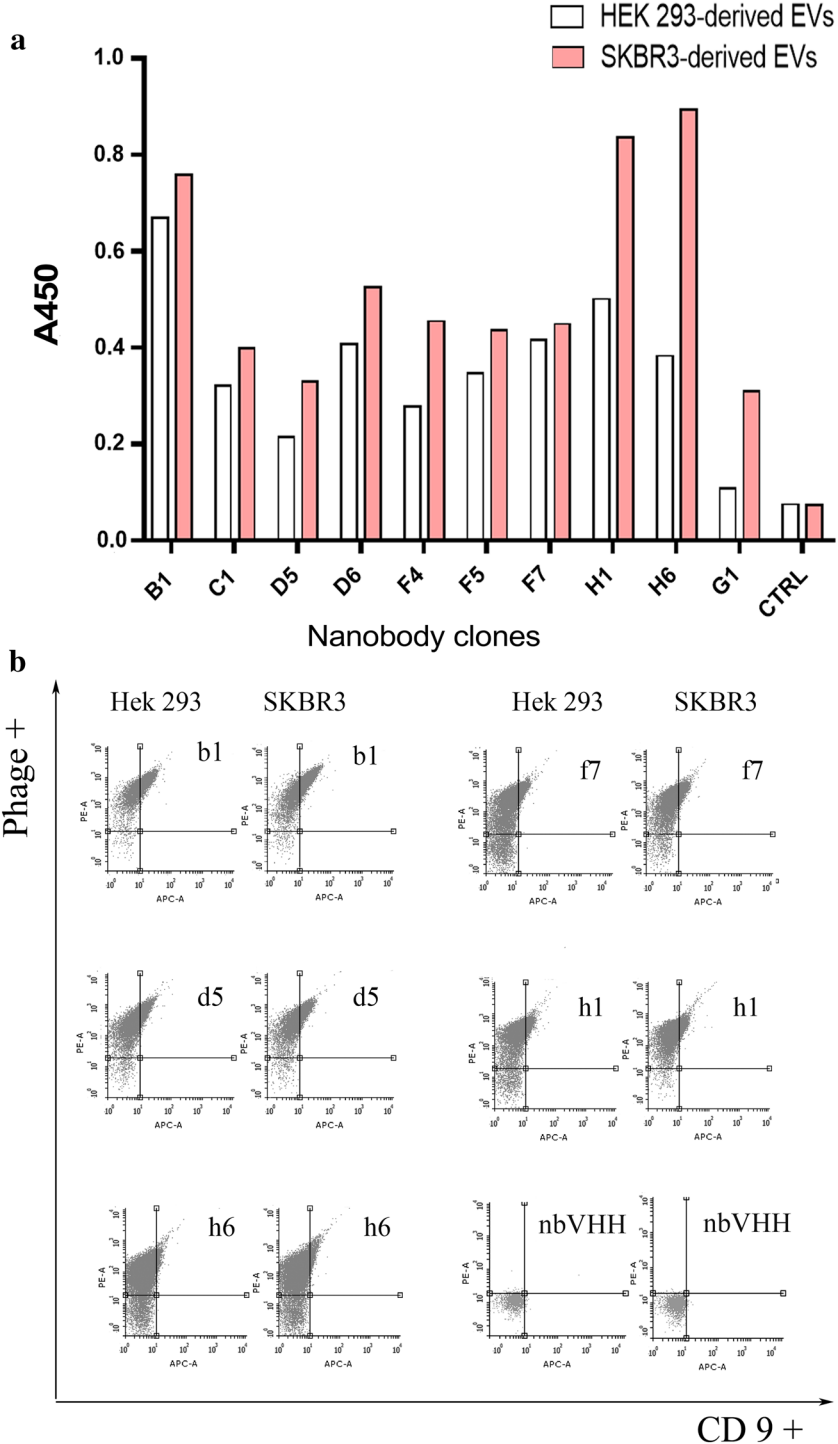
Binding characteristics of phage-displayed nanobodies isolated by direct panning on EV-enriched samples. **a** Phage-ELISA of clones selected by panning was performed to confirm their specific binding to EV-enriched fractions recovered from SKBR3 and HEK293 cells. Assay microplates were coated with EV-enriched samples (5 μg/well of protein) and bound phages were detected with HRP-labelled anti-M13 antibodies. An irrelevant phage-displayed nanobody was used as a negative control. **b** Characterization of phages binding to EVs by double-staining flow-cytometry. The vesicle fractions (positive for anti-CD9 APC labelled antibodies) were further incubated with the nanobody-displaying phages previously isolated. Bound phages were visualized after staining by the addition of anti-M13 monoclonal antibodies and goat anti mouse PE antibodies. Gates were set according to the values of autofluorescence of naked beads (not coated with EVs) and an irrelevant phage-displayed nanobody was used as a negative control

An effective method for isolating antibodies able to recognize an antigen discriminating between two very close cell types is the blind in vitro selection known as differential panning. A pre-immune library is first incubated with the control population and all the antibodies bound to the membranes are discharged (depletion step). The unbound fraction is then challenged with the target cells and the antibodies that remain in solution are discharged, whereas the bound antibodies are recovered after elution (enrichment step) [30]. The approach has the further advantage of selecting binders that recognize the native conformation of the antigen because the selection is performed directly on whole cells and not on recombinant protein. We argued that it could be possible to adapt the protocol used for cells to EVs. We used the EV-enriched fraction corresponding to peak 1 obtained from chromatography of HEK293 supernatant for depletion and the material from peak 1 obtained from chromatography of SKBR3 supernatant for enrichment. In both cases, EV-enriched samples were bound to magnetic beads to simplify the washing steps. After two rounds of panning, 92 clones were analysed by phage ELISA on EVs recovered from both SKBR3 and HEK293 cells (Fig. 2a). This screening method uses directly culture media enriched in secreted phages and, despite the fact that the phage quantity is not normalized among the samples, is sufficient to discriminate between negative (14) and positive clones (78) which recognized EVs from both cell lines, usually with a preference for one of them. We did not identify binders with exclusive specificity for SKBR3 and selected 10 clones with high signal among those that apparently bound better to SKBR3-derived EVs. The sequencing results indicated the presence of five unique sequences. These underwent further validation by flow-cytometry in combination with commercial antibodies against CD9 (Fig. 2b). The data indicated two distinct binding behaviours for the nanobody clones. Whereas the two antibodies seemed to bind independently when the commercial anti-CD9 was tested with B1, F7, and D5, in the presence of the clones H1 and H6 the binding of the anti-CD9 appeared to be inhibited. Since CD9 is one of the canonical EV biomarkers, showing that we selected anti-CD9 nanobodies would indicate that direct panning on EVs is not only feasible and yields functional binders, but that at least part of them are directed against EVs, despite the low purity of the material used for the panning [19]. A consideration is necessary here to explain the lack of SKBR3-selective binders. We imputed it to the elevated loss of EVs from the beads during the washing steps that we observed. Under these conditions the library depletion was probably insufficient for eliminating clones able to recognize shared epitopes.

### H1 and H6 VHH-GFP constructs compete with anti-CD9 antibodies

To prove that H1 and H6 were anti-CD9 antibodies, these clones were first sub-cloned in an expression vector that allows the production of fusion proteins composed of nanobody, eGFP, and C-term 6xHis tag. The preliminary small-scale expression test showed that the constructs were produced in soluble form in *E. coli* (data not shown) and was used to set the optimal growth conditions. Purification of VHH-GFP constructs (42 kDa) from soluble bacterial fractions by metal-affinity chromatography resulted in non-homogenous preparations (Additional file 2: Figure S2) which needed an anion-exchange chromatographic step to separate contaminants from the fluorescent immunoreagents (Additional file 2: Figure S2). Final yields were in the range of 10 mg of homogeneous VHH-GFP per litre of medium and these purified constructs were used in competition experiments at flow-cytometry. Binding comparison to EVs derived from different cell lines confirmed that some clones (H1 and B1) had clearly differing binding preferences, an indication that they recognize different antigens on the EVs (Additional file 3: Figure S3A–C).

**Fig. 3:**
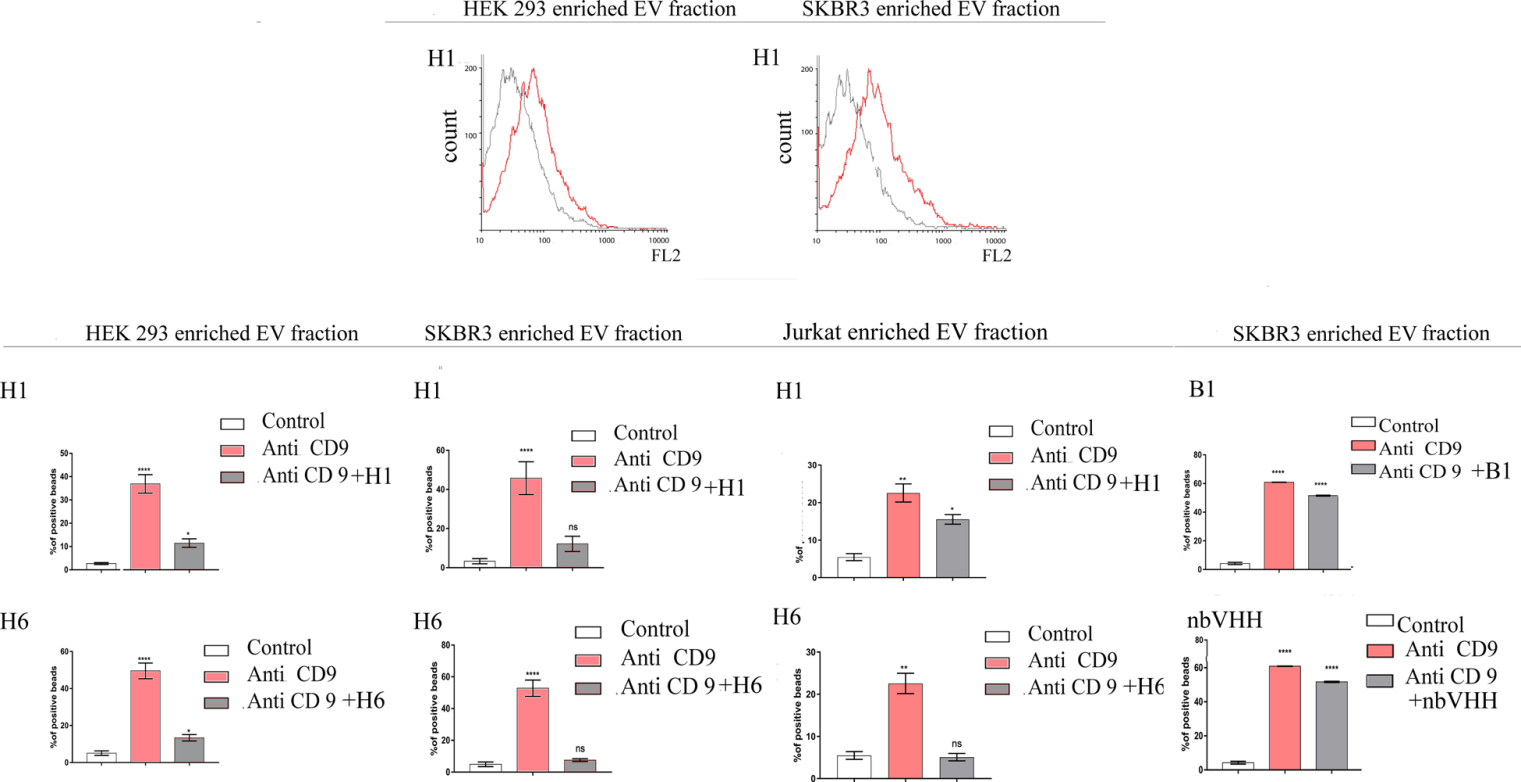
GFP-fused nanobodies H1 and H6 compete with anti-CD9 for EV epitopes. The binding of anti-CD9-PE to EVs (from either SKBR3 or HEK 293 cells) immobilized on latex beads was monitored by flow-cytometry in the presence of (gray) or absence of (red) the VHH-GFP constructs H1 (reported as an example), H6, B1 (CD9-neg/EV-pos clone), and nbVHH (irrelevant non-binding control nanobody). Latex beads are slightly autofluorescent (data not shown). The inhibition rate was calculated by analysis of variance, using the Kruskal–Wallis test followed by the Tukey’s post hoc test (p < 0.05). The error bars indicate standard deviations for triplicate measurements

Latex beads coated with EV-enriched fractions recovered from HEK293, SKBR3, and Jurkat cells were tested for their CD9 positivity with a commercial monoclonal antibody (Additional file 3: Figure S3D), then were incubated either with anti-CD9–PE antibodies or with both anti-CD9–PE and VHH-GFP antibodies (Fig. 3). As seen by the shift in FL2 fluorescence intensity, the addition of H1 nanobodies significantly decreased the binding of anti-CD9 to beads coated with EVs. This was confirmed with EVs derived from all the three cell types and the H6 clone. In terms of labelled beads (Fig. 3), the presence of H1-GFP inhibited the CD9 binding from 36.86 ± 9.95 to 11.46 ± 1.82% (HEK293-EVs) and from 45.78 ± 8.42 to 12.18 ± 3.89% (SKBR3-EVs), while H6-VHH competition resulted in a decrease of anti-CD9 bound beads from 49.55 ± 4.21 to 13.40 ± 1.73% (HEK293-EVs) and from 52.86 ± 5.14 to 7.59 ± 0.86% (SKBR3-EVs). Controls such as an irrelevant nanobody (nbVHH) and the B1 clone which was assessed as a CD9-neg but EV-pos in the preliminary screening (Fig. 2b) did not affect significantly the CD9 binding to the vesicle-coated beads (Fig. 3). The capacity of the monovalent VHHs to compete successfully with the bivalent commercial monoclonal IgG was observed at conditions of almost 200 times molarity excess for the nanobodies in solution.

### Nanobody-mediated EV capture on solid surfaces

**Fig. 4:**
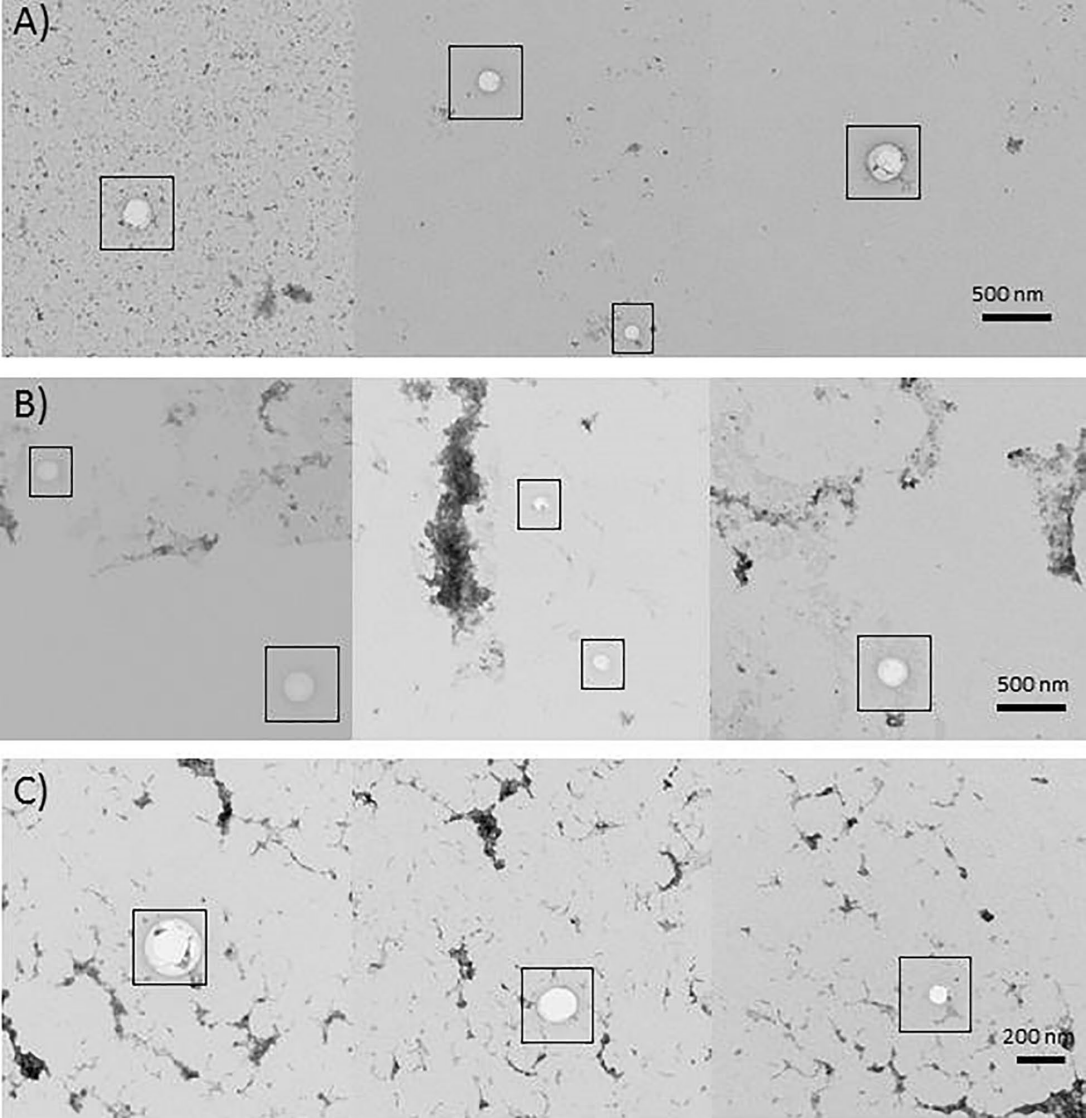
TEM analysis of immune-purified EVs. EVs recovered by either precipitation (**A**) or H1/H6-mediated immune-purification from cell culture supernatant (**B**) and human serum (**C**) were analysed after uranyl acetate negative staining. Three independent pictures are shown for each sample to show the EV dimension variability (50–200 nm)

The results of the competition experiments seemed to indicate that H1 and H6 are anti-CD9 nanobodies. This feature enables their use as specific binders for EVs for improving the stability of their binding (to beads, to ELISA plate, to biosensor surfaces, etc.) and their selective capture when contaminated EV fractions are available. As a proof-of-principle, H1/H6 nanobodies were used for immune-purification of EVs from both cell culture supernatant and human serum. The nanobodies were coated onto latex beads and used to bind the EVs present in the samples. EVs were then released by lowering the buffer pH. Beads were incubated with 20 µg of EV-fraction proteins recovered from supernatant of HEK293, SKBR3, and Jurkat cells. The yields (9–11 µg) were constant for all the samples. In the case of human plasma, immune-purification resulted in 42 µg of exosomal protein per 250 µL of undiluted plasma. A qualitative evaluation of the recovered EVs was performed by TEM (Fig. 4). This analysis identified vesicles with diameter between 50 and 200 nm in all the observed samples.

**Fig. 5:**
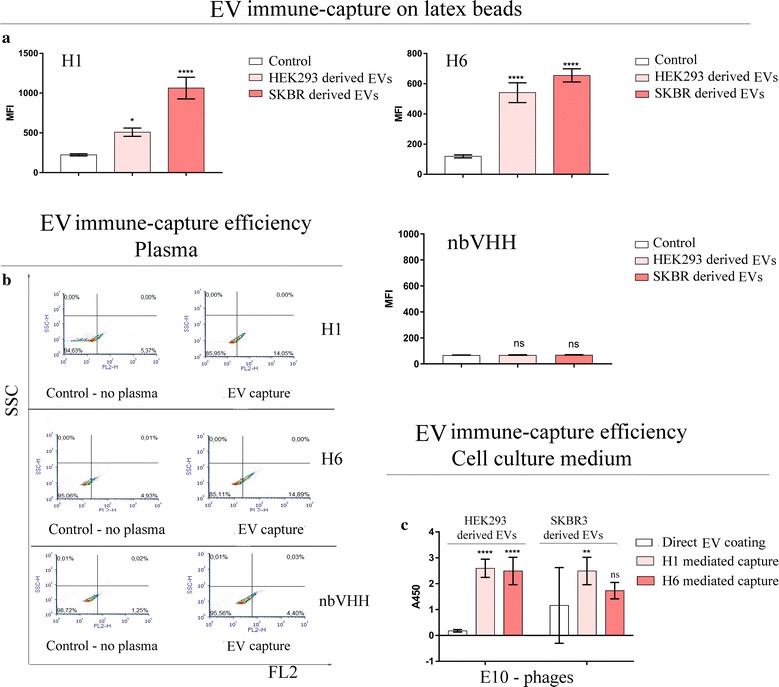
Sandwich assay for EV capture with VHH-GFP constructs tested by flow-cytometry and ELISA. Latex beads were coated with purified nanobodies, EVs were captured from cell culture and detected by flow-cytometry with anti-CD9-PE labelled antibodies. Bars indicate median fluorescence intensity of bound anti-CD9–PE antibodies to H1-GFP, H6-GFP, and irrelevant (EV-neg) VHH-GFP coated beads (**a**). Direct capture of EVs from plasma (**b**) was assessed by flow-cytometry using VHH-GFP coated beads. Anti-CD9-PE was used for detection and bead autofluorescence was measured in the absence of plasma (controls). **c** ELISA microplates were either directly coated with EV-enriched fractions or EVs were bound by means of previously immobilized purified anti-CD9 nanobodies. Such EV-prepared microplates were used to capture anti-EV nanobodies displayed on phages which were detected by adding anti-M13, HRP-labelled antibodies. The error bars indicate standard deviations for triplicate measurements

Next, we evaluated if the nanobody-mediated capture could increase the amount of EVs stably bound to the latex beads used for flow-cytometry. Both H1 and H6 coated beads were used in combinations with samples of cell culture supernatant collected from HEK293 and SKBR3 cells and the immune-captured EVs were used to bind anti-CD9–PE antibodies. The amount of captured EVs was finally assessed indirectly by measuring the PE-dependent fluorescence intensity (Fig. 5a). A clear increase was measured for both tested nanobodies, with constant higher fluorescence values obtained when using material from SKBR3 cell cultures. Both nanobodies bound to latex beads were successful in capturing EVs also from human plasma of healthy donors (Fig. 5b). Despite the elevated background signal and the low concentration of EVs in these samples [36], the fluorescence value increased from 5.37% (control) to 14.05% (H1-coated beads) and from 4.93% (control) to 14.89% (H6-coated beads). An irrelevant nanobody (nb-VHH) used as a control was not able to capture vesicles from human plasma. Purified H1 and H6 nanobodies were also able to bind strongly to the fraction 1 separated by IEX chromatography and linked to latex beads (Additional file 4: Figure S4), in agreement with the data previously obtained with monoclonal anti-CD9 that confirmed the presence of EVs in this sample.

Next we assessed the possibility to improve the reproducibility of EV ELISA by using H1- and H6-mediated nanobody immune-capture instead of direct EV coating on microplate surface to reduce the vesicle removal during the washing steps. In the experimental setting, EVs from cell culture supernatant were first immobilized in the microplate wells – directly or by means of anti-CD9 nanobodies-and later used to screen phage displayed nanobodies according to their affinities for such EVs (Fig. 5c). Bound phages were visualized using anti-M13 HRP antibodies. The false negative (EVs from HEK293 cells) and the high standard deviation (EVs from SKBR3) values obtained by direct EV coating indicated highly unstable conditions and/or consequent low data reproducibility. H1/H6-mediated EV immune-capture eliminated the variability and enabled to collect reproducible data. Probably, EVs directly coated on the microplate plastic are apparently prone to be washed away and adopting this methodology resulted in misleading results. In detail, the absorbance (A_450_) values describing the E10 clone binding capacity to HEK293-derived EVs passed from 0.18 ± 0.05 (direct coating) to 2.60 ± 0.35 and 2.49 ± 0.53 for H1- and H6-mediated EV capture, respectively. In the case of the SKBR3 EVs, direct coating yielded an A_450_ value of 1.16 ± 1.46, whereas nanobody-mediated EV coating resulted in A_450_ values of 2.49 ± 0.53 and 1.73 ± 0.31.

## Discussion

The involvement of EVs in both physiological and pathological processes [1] makes them promising diagnostic and prognostic biomarkers [12, 22]. In the last years research aimed at standardizing the EV purification and characterization methods as well as stratifying EV sub-populations for improving the reliability of the diagnostic information. This development led to an increasing interest for affinity-, and specifically for immune-affinity-based purification methods. However, the elevated development and production costs of reagents, such as monoclonal antibodies suitable for specific EV biomarkers, slowed down this approach implementation. In this work we clearly demonstrate the feasibility of isolating nanobodies by panning directly against EV-enriched fractions. This methodology is fast and inexpensive in comparison to conventional hybridoma protocols (Additional file 5: Table S1). Furthermore, it presents two additional major advantages: (i) the selected binders recognize EV-epitopes in their native conformation; (ii) potentially, it enables to isolate binders against new biomarkers. The first benefit was directly exploited in the present work. We had noticed that the major drawback of direct panning on EVs was due to the poor binding of the vesicles to solid surfaces, namely latex beads during the panning and plastic microplates during ELISA. This condition impaired the application of a successful differential panning to isolate nanobodies which can discriminate between subgroups. When we used the two selected nanobodies which apparently compete with CD9 (Fig. 3) for the immunocapture of the vesicles to the solid surfaces, EV binding was strongly stabilized and experimental variability was drastically reduced (Fig. 5). This is a critical accomplishment since it allows for the standardization and reproducibility of the selection procedure and will enable the implementation of differential panning protocols to fish for nanobodies that can discriminate between exosome sub-populations, as we already successfully performed with whole cells [30]. It is probable that an accurate EV profiling will require the analysis of a large amount of both qualitative and quantitative biomarkers [37, 38], namely of several corresponding antibodies. As already demonstrated in this work with the constructs VHH-GFP, the straight-forward bacterial production of immune-fluorescent fusions can provide inexpensive application-friendly reagents [27] for multi-dimensional vesicle characterization. These immune-reagents can be directly used with complex samples such as human plasma without any pre-fractionation (Fig. 5b).

In this work we also showed that anion-exchange chromatography can be used for preliminary separation of EV fractions. The presence of several fractions demonstrates the heterogeneity of vesicular content and existence of several sub-populations even in a simplified system such as conditioned cell culture supernatant. Large preparative anion-exchange purification could provide the material necessary for: (i) the characterization of the molecular content of the different fractions, both in terms of EV sub-types and “contaminants” such as soluble proteins; (ii) performing differential panning and isolation of binders which selectively recognize the EV fraction present in only one elution peak or distinguish the same peak in material obtained from different samples, discriminating between clinical relevant and non-relevant markers. Advantages of monolithic supports for ion chromatography include better mass transfer properties, the ability to be manufactured with a wide range of pore sizes, the relative ease of scaling up and scaling down, and the low back pressure even at very high flow [39, 40]. Monoliths can be functionalized to perform different separation modes [41, 42], including immune-affinity, and therefore should be further considered for miniaturized multi-components for EV-based diagnostics [43].

## Conclusions

In this work we report the first case of successful isolation of anti-EV nanobodies by direct panning of a phage library on partially purified EVs. This achievement enables stable immunoaffinity-based EV capture and consequently simplifies the future discovery of novel antibody-vesicle surface biomarker pairs that will be instrumental for the systematic stratification of EV sub-populations and their individual characterization.

## Methods

### Cell culture

Human embryonic kidney (HEK-293), breast adenocarcinoma (SKBR3), and T cell lymphoma (Jurkat) cells were obtained from the American Type Culture Collection (ATCC) (Rockville, MD, USA). HEK-293 and SKBR3 cells were grown in Dulbecco’s modified minimal essential medium (DMEM) supplemented with 10% v/v heat inactivated fetal bovine serum (FBS), 100 U/mL penicillin and 100 µg/mL streptomycin (Gibco, Thermo-Fisher Scientific) at 37 °C under a 5% CO_2_/95% air atmosphere at constant humidity. Jurkat cells were grown in RPMI medium supplemented with 10% v/v heat inactivated fetal bovine serum (FBS), 100 U/mL penicillin and 100 µg/mL streptomycin (Gibco, Thermo-Fisher Scientific) at 37 °C under a 5% CO_2_/95% air atmosphere at constant humidity.

### Purification of EV-enriched fractions from cell culture supernatant

SKBR3 and HEK293 cells were grown in DMEM medium supplemented with FBS (not EV-free) until they reached 70–80% confluence. Jurkat cells were grown in RPMI medium supplemented with FBS (not EV-free) for 3 days. The culture medium was then removed and the cells rinsed with PBS before the addition of FBS-free DMEM medium in the case of HEK-293 and SKBR3, or FBS-free RPMI in the case of Jurkat cells. After 24 h, the conditioned cell culture supernatant was transferred into 50 mL polypropylene tubes and centrifuged 30 min at 300×*g* at 4 °C to pellet floating cells and filtered through 0.45 µM syringe filter to remove cell debris and aggregates. The supernatant was collected into a sterile glass bottle and used for chromatographic separation using an ÄKTA pure 25 system (GE Healthcare) in combination with a 1 mL CIM QA-1-monolith column with pores 6 µm in diameter (BIAseparations) equilibrated in 20 mM Tris–HCl, pH 8.03. Two millilitre of exhausted media were used, corresponding to a culture of roughly 1 million cells, diluted 1:10 in 20 mM Tris–HCl buffer, pH 8. After a 10 mL washing step with the equilibration buffer, a 20 mL linear salt gradient (0–100%) was obtained by adding 20 mM Tris–HCl, pH 8.03, 2 M NaCl at a flow rate of 5 mL/min. The eluted material was monitored detecting the absorbance at 280 nm. As an alternative, an EV-enriched fraction was purified using the Total Exosome Isolation kit (Life Technologies, Thermo-Fisher Scientific), according to the manufacturer’s instruction and ultrafiltration using an ultra-centrifugal cartridge with cut-off of 100 kDa (Amicon, Merck).

### Direct panning on EV-enriched fractions

Panning was performed following a modified version of previously described methods [29]. Nanobody-displaying phages (3 × 10^11^)—diluted in 1 mL of PBS containing 2% skimmed milk—were first depleted by incubating them twice 30 min in the presence of 25 µL of milk-blocked naked beads and each time the bound fraction was discharged. The unbound phage fraction was transferred to the HEK293 EV-coated beads and incubated for 1 h. EV-coated beads were prepared using epoxy magnetic beads (25 µL; Life Technologies, Thermo-Fisher Scientific). Epoxy magnetic beads were washed in PBS and finally resuspended in 25 µL of the same buffer before adding 2 µg of the EV-enriched fraction recovered by chromatographic separation of the material present in HEK293 cell culture supernatant. After overnight incubation, the still reactive sites on bead surfaces were blocked 30 min at room temperature with 1 mL of PBS, 2% milk powder. Pre-selected phages were incubated with EV-coated beads for 1 h at room temperature and the unbound fraction was recovered. The depletion procedure as repeated and finally the unbound phage fraction was incubated with beads coated with EV-enriched fraction recovered by chromatographic separation of SKBR3 culture media (see above). After 20 washing cycles in PBS, bound phages were eluted by adding 1 mL of 200 mM glycine, pH 2.2 containing 1 mg/mL BSA and neutralized in 150 µL of 1 M Tris–HCl, pH 9.1. The phages were used to infect TG1 cells that were spread over 2xTY Petri dishes. The resulting colonies were recovered the successive day and the corresponding rescued phages used for a second panning cycle.

### Ev elisa

The phages produced by the colonies recovered after the second panning cycle were screened by ELISA for their capacity to bind EVs. An irrelevant clone was used as a negative control. Microtiter plates (96-well, MaxiSorp, Nunc™) were coated by incubating overnight at 4 °C with 2 μg/well (100 µL/well in PBS) of total protein from EV-enriched fractions. Plates were blocked (1 h at 37 °C in PBS buffer plus 5% (w/v) skimmed milk) and phages pre-incubated 1 h at 37 °C in PBS buffer plus 1% (w/v) skimmed milk. One hundred microliter per well of blocked phages were added to the plates and incubated 1 h at room temperature. After washing (4 times × 5 min in PBS), 100 μL of mouse anti-M13 antibodies conjugated with HRP (dilution 1:5000, GE Healthcare) were added to each well and incubated 1 h at 37 °C. Reporter signal was obtained by adding 100 µL of TMB solution (Sigma Aldrich) after another washing step (4 times × 5 min in PBS). The colour reaction was stopped by 50 μL of 2 NaH_2_SO_4_. Absorbance at 405 nm was recorded by using a HTS7000 Bioassay reader (Perkin Elmer).

### Nanobody subcloning and production

Nanobody sequences were subcloned into a modified pET14b vector using *Nco*I and *Not*I enzymes to obtain fusion constructs in which nanobodies are linked at their C-terminal with both eGFP and 6xHis tag [27]. These vectors were transformed into *E. coli* BL21 (DE3) hosting the plasmid for the expression of sulfhydryl oxidase and DsbC [44, 45]. Antibodies were produced as previously described with some modifications [27]. Briefly, 2 mL of over-night pre-culture were used to inoculate 500 mL of LB broth in the presence of the 100 µg/mL ampicillin and 25 µg/mL chloramphenicol. Bacteria were grown at 37 °C until OD_600nm_ reached 0.4. Sulfhydryl oxidase and DsbC expression was induced by adding 0.5% (g/mL culture) of arabinose and the temperature was lowered to 30 °C. After 30 min, 0.1 mM of IPTG was added to induce antibody expression, the bacteria were grown overnight at 21 °C, harvested, and frozen. Bacterial pellet was resuspended in 20 mL of 100 mM Tris–HCl, pH8, 500 mM NaCl, 2.5 mM MgCl_2_. Lysozyme (0.5 mg/mL) and DNAse (3U) were added and the lysate was kept 30 min at room temperature. Samples were sonicated and finally centrifuged at 18,000×*g* for 20 min at 4 °C. Supernatant was filtered (0.45 µM) and loaded on a 5 mL HiTrap Talon Crude column (GE Healthcare) connected to a chromatographic ÄKTA pure 25 system (GE Healthcare). The column was equilibrated with 50 mM phosphate buffer, pH 7.4, containing 500 mM NaCl and 5 mM imidazole, while bound proteins were eluted using 50 mM phosphate buffer, pH 7.4, containing 500 mM NaCl and 150 mM imidazole. Fractions containing VHH-GFP constructs were pooled and buffer was exchanged into 30 mM Tris–HCl buffer, pH 8.3, by using a 5 mL HiTrap Desalting column (GE Healthcare) in combination with a chromatographic ÄKTA pure 25 system (GE Healthcare). Further purification of the VHH-GFP construct was achieved using a Mini Q 4.6/50 ion exchange column (GE Healthcare) mounted on a chromatographic ÄKTA purifier ten system (GE Healthcare). Samples were loaded on a column pre-equilibrated with loading buffer (30 mM Tris buffer, pH 8.3) and eluted using 30 mM Tris buffer, pH 8.3, 1 M NaCl. Elution was achieved by using a linear 0–100% gradient (10 column volumes). Antibody concentration was determined by Bradford colorimetric assay [46] while the presence of contaminants was assessed by SDS-PAGE (14%, denaturing conditions).

### Flow-cytometry of EV fractions

Flow-cytometry was performed by using either phages or purified nanobodies. In the first case, the protocol started with the coating of aldehyde/sulphate latex beads 4% w/v, 4 µm (Thermo Fisher Scientific) with EVs. Specifically, 25 µL of latex beads were coated overnight at 4 °C with 2 µg of total protein from EV-enriched fraction in PBS. Beads were washed 3 times with PBS and blocked first for 30 min at room temperature with 200 mM glycine and then 30 min in PBS plus 5% (w/v) skimmed milk. Phages were pre-incubated 1 h at 37 °C in PBS buffer plus 1% (w/v) skimmed milk and 100 µL of the blocked phages were incubated with 25 µL beads for 45 min at room temperature. Beads were washed 3 times in PBS before adding 100 μL of mouse anti-M13 antibodies (dilution 1:100, GE Healthcare). After 30 min at room temperature, beads were washed 4 times in PBS and 100 μL of anti-mouse PE-labelled antibodies (dilution 1:100, BD Biosciences) were added. After 30 min at room temperature and another washing step as above, 100 μL of anti-CD9 APC-labelled antibodies (BD-Biosciences, dilution 1:100, final concertation 16.7 nM) were added. Beads were incubated 20 min at room temperature before washing and flow-cytometry analysis by using a FACSCanto II instrument (BD Biosciences) collecting around 1000 events/s. A blue solid state 200 mW laser at 488 nm and a 100 mW red laser at 640 nm were used for excitation. The emission was detected with 561 nm (FL2, PE) and 660 nm (FL3, APC) filter. The positive beads were gated on the FL2-PE, FL3-APC plot. APC and PE specific fluorescence was assessed as the signal increase with respect to negative control (autofluorescence of beads without coated EVs).

When purified VHH-GFP constructs were used for detection of EVs, 25 µL of aldehyde/sulfate latex blocked beads containing EVs were incubated with 100 µg of nanobodies (2.5 μM) in 1% BSA solution in PBS for 1 h at room temperature. Beads were washed 3 times in PBS before adding 100 μL of mouse anti-CD9 PE-labelled antibodies (dilution 1:100, GE Healthcare) and were then incubated for 45 min at room temperature. After a washing step as described before, beads were analysed using FACS Calibur (BD Biosciences). A blue solid state 200 mW laser at 488 nm was used for excitation. The emission was detected with 525 nm (FL1, GFP) and 561 nm (FL2, PE) filter. The positive beads were gated on the FL1-GFP, SSC plot and FL2-PE, SSC plot. GFP and/or PE specific fluorescence was assessed as the signal increase with respect to negative control (autofluorescence of beads without coated EVs).

### Capture of EVs on solid phase using purified nanobodies

EVs were captured using purified VHH-GFP constructs in both ELISA and flow-cytometry format assays. For ELISA, microtiter plates (96-well, MaxiSorp, Nunc™) were coated by incubating 10 μg/well of VHH-GFP (100 µL/well in PBS) overnight at 4 °C. Plates were blocked (1 h at 37 °C in PBS buffer plus 5% (w/v) skimmed milk) after which 0.1 µg of total protein from EV fraction in PBS with 1% milk (w/v) was added and incubated 1 h at 21 °C. After washing (4 × 5 min, PBS), 100 µL/well of blocked phages were added to the plates and incubated 1 h at room temperature. After washing (4 × 5 min in PBS), 100 μL of mouse anti-M13 antibodies conjugated with HRP (dilution 1:5000, GE Healthcare) were added to each well and incubated 1 h at 37 °C. Signal was developed after another washing step by the addition of 100 µL of TMB solution (Sigma Aldrich) and 1 h incubation at 37 °C. The colour reaction was stopped by 50 μL of 2 NaH_2_SO_4_. Absorbance was monitored at 405 nm in a HTS7000 Bioassay reader (Perkin Elemer).

For flow-cytometry, 25 µL of latex beads were coated overnight at 4 °C with 50 µg of VHH-GFP. Beads were washed three times with PBS and blocked first for 30 min at room temperature with 200 mM glycine and then 30 min in PBS plus 5% (w/v) skimmed milk. Beads were incubated with 10 µg of total protein from EV enriched fraction in PBS with 1% milk (w/v)/10 µL of VHH-coated beads or with 1:2 diluted plasma pool from healthy volunteers for 1 h at room temperature. Beads were washed 3 times in PBS before adding 100 μL of mouse anti-CD9 PE-labelled antibodies (dilution 1:100, GE Healthcare) and incubated for 45 min at room temperature. After washing steps as described before, beads were analysed using FACS Calibur (BD Biosciences). The analysis rate was around 1000 events s-1. A blue solid state 200 mW laser at 488 nm was used for excitation, the emission was detected at 561 nm (FL2, PE). The positive beads were gated on the FL2-PE, SSC plot. Specific fluorescence was assessed as the signal increase with respect to negative control (autofluorescence of beads without coated EVs).

### EV immune-purification and TEM analysis

Twenty microliter of latex beads were coated overnight at 4 °C with 100 µg of H1/H6-GFP. Beads were washed three times with PBS and blocked first for 30 min at room temperature with 200 mM glycine and then 30 min in PBS plus 5% (w/v) skimmed milk. Beads were incubated 1 h at room temperature with: (i) 20 µg of total protein from cell supernatant EV enriched fraction in PBS with 1% milk (w/v); (ii) plasma pool from healthy volunteers diluted 1:2 in the same buffer. Beads were washed 5 times in PBS before elution in 100 µL of 200 mM glycine, pH 2.2 and immediate neutralization in 15 µL of 1 M Tris–HCl, pH 9.1. Eluted vesicles were stabilized by the addition of 25 mM trehalose and their morphology was evaluated by TEM after sample negative staining. Four microliter of vesicle suspension were adsorbed on carbon/formvar coated 400 mesh nickel grids (Electron Microscopy Sciences, Fort Washington, USA). After 10 min, the sample excess was removed with filter paper and immediately replaced by 4 μL of staining agent (uranyl acetate diluted 1:3 in distilled water), which was allowed to settle 10 min. Grids were washed 5 times in distilled water, the water excess was removed with filter paper, and then observed in a Philips CM 10 (FEI, Eindhoven, The Netherlands) TEM, operated at 80 kV.

### Statistical analysis

Statistical analysis was performed using GraphPad Prism v7 for Windows (San Diego). A significance level of p ≤ 0.05 was used for analysis of variance, implemented using the Kruskal–Wallis test followed by the Tukey’s post hoc test (p ≤ 0.05). Correlation between different parameters was performed at significance level of p ≤ 0.05.

## Additional files

**Additional file 1: Figure S1.** **Chromatographic separation of kit-purified EV-enriched fraction from culture media.** Kit-precipitated EVs present in SKBR3 cell culture supernatant were separated using a large-pore anion-exchange monolith column. All the four separated fractions were analyzed by flow-cytometry and resulted positive for the EV marker CD9.

**Additional file 2: Figure S2.** **Purification strategy of VHH-GFP constructs.** H1-GFP and H6-GFP constructs were expressed in *E. coli* and purified from the soluble fraction using immobilized metal-affinity chromatography. The corresponding chromatograms are reported together with the SDS-gels of total bacterial lysate (lane 1) and elution fractions 10–12 (lanes 2, 3, 4). After desalting, the samples underwent IEX purification and the eluted fractions were separated by SDS-PAGE (H6, lanes 1–10; H1, lanes 11–19; *MW* molecular weight markers).

**Additional file 3: Figure S3.** **Antibody differential binding to cell-derived EVs.** EVs derived from HEK-293, SKBR3, and Jurkat cells were used to evaluate the binding preferences of the nanobodies H1 and B1 compared with the binding of the irrelevant clone nbVHH (**A**–**C**). The binding capacity of a commercial anti-CD9 antibody was tested with the same cell lines (**D**). Bars indicate median percentage of positively stained EV coated beads with anti-CD9–PE antibodies to H1-GFP, B1-GFP, and non-binding VHH coated beads with respect to autofluorescence of unstained EV coated beads. The error bars indicate standard deviations for triplicate measurements.

**Additional file 4: Figure S4.** **Anti-exosome nanobodies bind EV-fractions separated by chromatography.** Flow cytometry experiments show that both H1 and H6 strongly bind to exosomes present in the fraction 1 separated by IEX chromatography.

**Additional file 5: Table S1.** Comparison of indicative times and costs necessary to produce monoclonal antibodies.
