# Supplementary figures and images for: Isolation of anti-extra-cellular vesicle single-domain antibodies by direct panning on vesicle-enriched fractions
Source: Microb Cell Fact. 2018 Jan 13;17:6. doi: 10.1186/s12934-017-0856-9 (PMC5766977; doi:10.1186/s12934-017-0856-9)

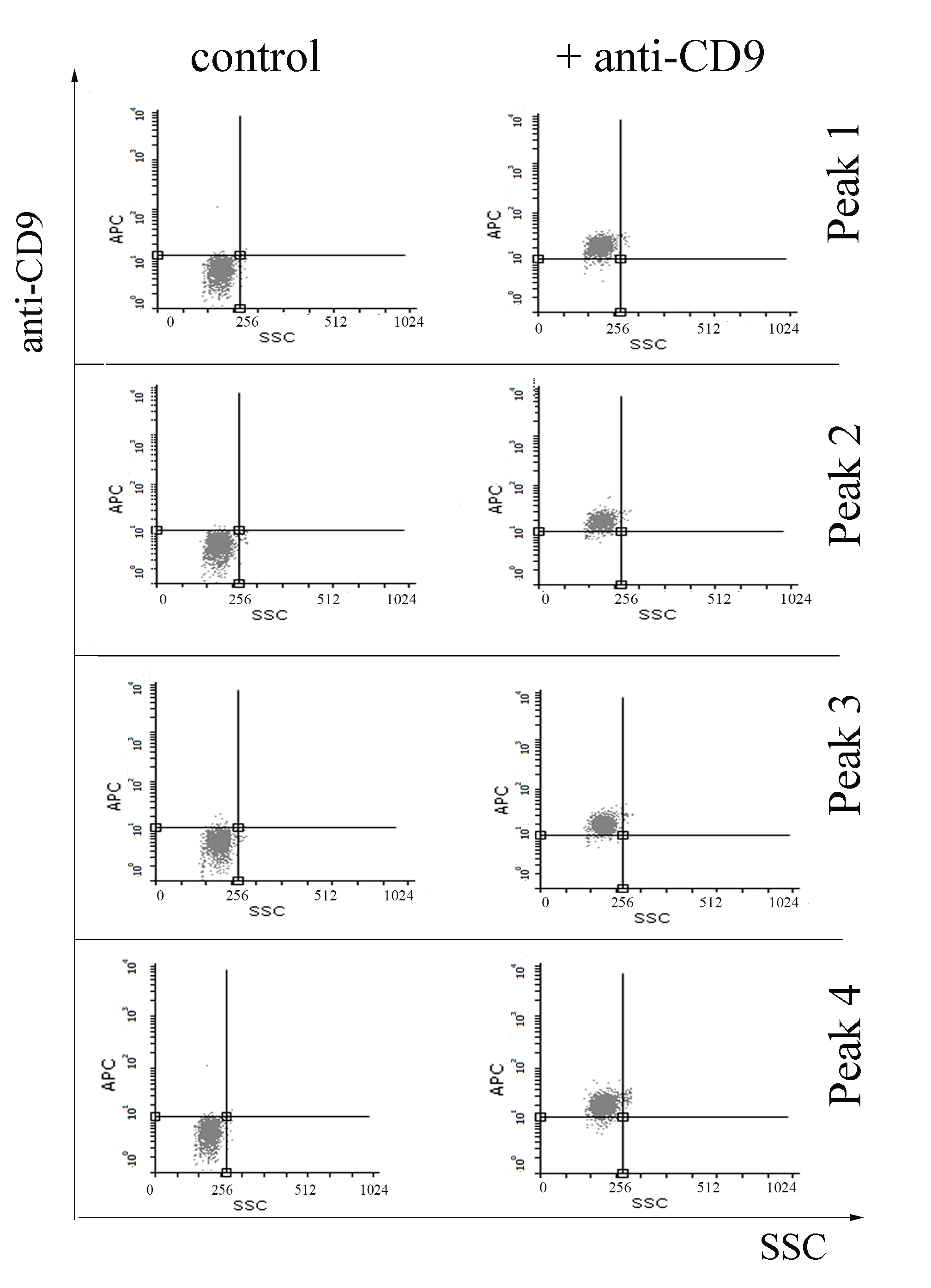

Supplement: Supplementary file 1 — Additional file 1: Figure S1. Chromatographic separation of kit-purified EV-enriched fraction from culture media. Kit-precipitated EVs present in SKBR3 cell culture supernatant were separated using a large-pore anion-exchange monolith column. All the four separated fractions were analyzed by flow-cytometry and resulted positive for the EV marker CD9. [file 12934_2017_856_MOESM1_ESM.tif]

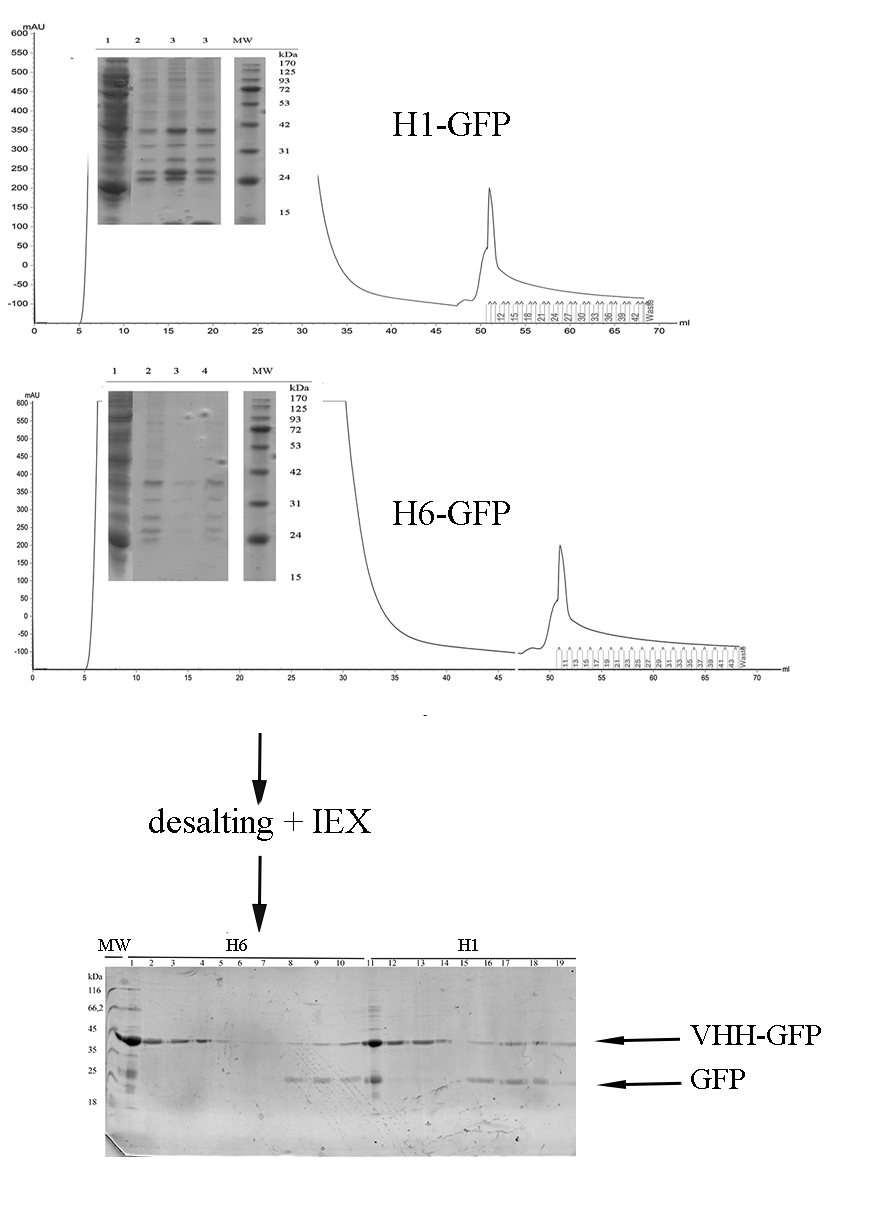

Supplement: Supplementary file 2 — Additional file 2: Figure S2. Purification strategy of VHH-GFP constructs. H1-GFP and H6-GFP constructs were expressed in E. coli and purified from the soluble fraction using immobilized metal-affinity chromatography. The corresponding chromatograms are reported together with the SDS-gels of total bacterial lysate (lane 1) and elution fractions 10–12 (lanes 2, 3, 4). After desalting, the samples underwent IEX purification and the eluted fractions were separated by SDS-PAGE (H6, lanes 1–10; H1, lanes 11–19; MW molecular weight markers). [file 12934_2017_856_MOESM2_ESM.tif]

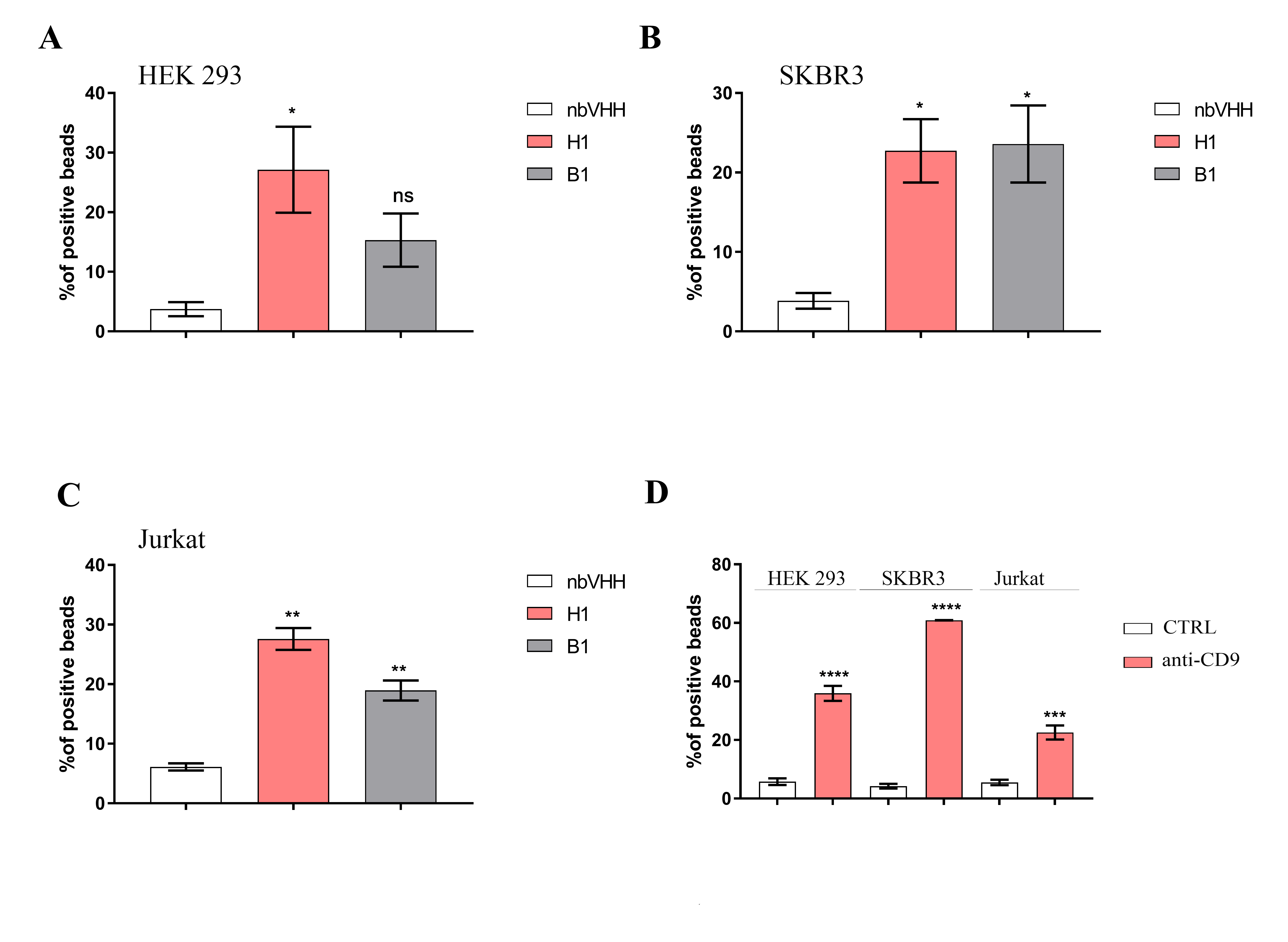

Supplement: Supplementary file 3 — Additional file 3: Figure S3. Antibody differential binding to cell-derived EVs. EVs derived from HEK-293, SKBR3, and Jurkat cells were used to evaluate the binding preferences of the nanobodies H1 and B1 compared with the binding of the irrelevant clone nbVHH (A–C). The binding capacity of a commercial anti-CD9 antibody was tested with the same cell lines (D). Bars indicate median percentage of positively stained EV coated beads with anti-CD9–PE antibodies to H1-GFP, B1-GFP, and non-binding VHH coated beads with respect to autofluorescence of unstained EV coated beads. The error bars indicate standard deviations for triplicate measurements. [file 12934_2017_856_MOESM3_ESM.tif]

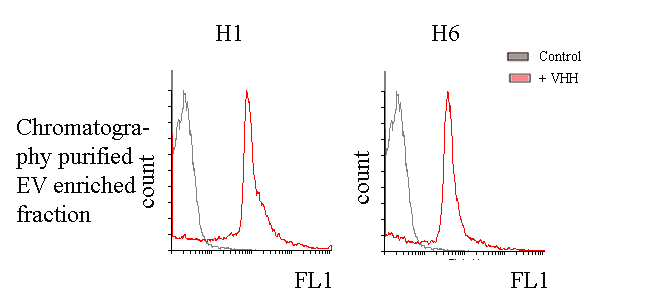

Supplement: Supplementary file 4 — Additional file 4: Figure S4. Anti-exosome nanobodies bind EV-fractions separated by chromatography. Flow cytometry experiments show that both H1 and H6 strongly bind to exosomes present in the fraction 1 separated by IEX chromatography. [file 12934_2017_856_MOESM4_ESM.tif]
