# Supplementary material for: Isolation of anti-extra-cellular vesicle single-domain antibodies by direct panning on vesicle-enriched fractions
Source: Microb Cell Fact. 2018 Jan 13;17:6. doi: 10.1186/s12934-017-0856-9 (PMC5766977; doi:10.1186/s12934-017-0856-9)
Supplement: Supplementary file 5 — Additional file 5: Table S1. Comparison of indicative times and costs necessary to produce monoclonal antibodies. [file 12934_2017_856_MOESM5_ESM.doc]

**Additional file: Table 1. Comparison of indicative times and costs necessary to produce monoclonal antibodies**

Calculations have been performed considering already available soluble antigens and material and personnel costs as experienced in our lab. In the case of membrane proteins, both time and costs can be significantly increased when the antigen must be purified for animal immunization. In contrast, there are no longer panning time and further costs for *in vitro* selection.

|  | **Monoclonal recombinant antibodies obtained by panning pre-immune libraries** | **Monoclonal antibodies obtained by hybridoma technology** |
| --- | --- | --- |
| **Time (immunization, Ab isolation and production)** | **1 month** | **4-6 months** |
| **Costs for Ab isolation** | **1000 euro** | **6-8000 euro** |
| **Costs for Ab production** | **20 euro/mg** | **1000-2000 euro mg** |

**Supplementary Figures**

**Supplementary Figure 1.** Chromatographic separation of kit-purified EV-enriched fraction from culture media

Kit-precipitated EVs present in SKBR3 cell culture supernatant were separated using a large-pore anion-exchange monolith column. All the four separated fractions were analyzed by flow-cytometry and resulted positive for the EV marker CD9

**Supplementary Figure 2**. Purification strategy of VHH-GFP constructs

H1-GFP and H6-GFP constructs were expressed in *E. coli* and purified from the soluble fraction using immobilized metal-affinity chromatography. The corresponding chromatograms are reported together with the SDS-gels of total bacterial lysate (lane 1) and elution fractions 10-12 (lanes 2, 3, 4). After desalting, the samples underwent IEX purification and the eluted fractions were separated by SDS-PAGE (H6, lanes 1-10; H1, lanes 11-19; MW: molecular weight markers)

**Supplementary Figure 3**. Antibody differential binding to cell-derived EVs

EVs derived from HEK-293, SKBR3, and Jurkat cells were used to evaluate the binding preferences of the nanobodies H1 and B1 compared with the binding of the irrelevant clone nbVHH (A-C). The binding capacity of a commercial anti-CD9 antibody was tested with the same cell lines (D). Bars indicate median percentage of positively stained EV coated beads with anti-CD9–PE antibodies to H1-GFP, B1-GFP, and non-binding VHH coated beads with respect to autofluorescence of unstained EV coated beads. The error bars indicate standard deviations for triplicate measurements

**Supplementary Figure 4.** Anti-exosome nanobodies bind EV-fractions separated by chromatography

Flow cytometry experiments show that both H1 and H6 strongly bind to exosomes present in the fraction 1 separated by IEX chromatography
